# Economy in the Generation and Use of Steam

**Published:** 1914-06-13

**Authors:** 


### ECONOMY IN THE GENERATION AND USE OF STEAM.

### II.—The Principal Apparatus Described.

### THE MECHANICAL STOKER.

Mechanical stokers are of two kinds: the "Overfeed" and the "Underfeed." With both forms coal is delivered to receptacles, from which it is discharged in definite quantities on to the grate; and the grate itself is kept in motion. In one form of the "Overfeed" stoker the coal is sprinkled on to the grate; while in another form definite quantities are lodged on the "dead plate," just inside the fire doors. In one kind of "Overfeed" stoker, again, the fire bars are given a jerking motion periodically; while in another form the grate itself is an endless chain of very large links, which is kept continually in motion. With the "Underfeed" stoker the coal is forced up between the fire bars, and moves gradually away from the point of entry. In all cases the fire doors are very rarely opened, and very inferior qualities of coal can be used. With one special form of stoker even very fine dust, known as "breeze," is used successfully and economically.

### Mechanical Draught.

Mechanical draught may be either "forced" or "induced." In both cases a fan driven either by a small engine, or more frequently by an electric motor, forces or sucks a current of air into the ashpit, and through the fire bars and the fuel, the current of air being regulated by the speed of the fan. With "forced" draught, either the boiler-room is closed and the air is forced into it, thence finding its way into the ashpit, or the ashpits are closed and air pipes lead to them. With "induced" draught the fan is placed at the back of the furnace, and has to be sufficiently large to accommodate the large volume of hot gas that is created by the combustion of the coal.

The feed water is heated in two ways: by the heat from the flue gases on their way to the chimney, or by the heat of the steam on its way to the atmosphere or to the condenser. The flue gases impart the largest amount of heat, but it is necessary that the feed water entering the "Economiser," as the apparatus in which the flue gases are made use of is called, should be raised to a temperature of about 90° F. before entering. The "Economiser" consists of a number of pipes arranged in a vertical stack and built into a chamber. The hot gases flow all over the outer surfaces of the pipes, and the water to be heated flows through the pipes. The hot flue gases deposit soot upon the pipes, and this has to be continually removed by means of scrapers supplied with the apparatus. The scrapers are kept continually working up and down the outside of the pipes, motion being imparted to them by a small engine or an electric motor.

In the other method of raising the temperature of water an apparatus called a "Feed Water Heater" is employed. This apparatus is very similar to the "calorifier" used for heating the water for use in the hospital and for the hot-water radiators. It consists usually of a cylinder with a nest of pipes inside. The water to be heated may pass through the pipes, while the steam flows around them; or the reverse arrangement may rule. The "Feed Water Heater" is usually placed between the condenser, when one is employed, and the "Economiser." In some forms of condenser a feed water heater is included in the apparatus, the steam from the engine passing over the pipes through which the feed water is flowing, before entering the condenser proper.

In all apparatus of this kind, where steam is employed, a certain amount of condensation takes place; and it is one of the economies of modern steam plant that all the condensed water shall be trapped and led away to a tank in any convenient place, from which it can be pumped to feed the boilers.

The condenser is almost a necessary part of any steam-power plant. It cannot always be employed in towns, because of the heavy cost of the cooling water. If the steam which has done its work in a steam-engine, say, driving dynamos for lighting and power or driving any other apparatus about the place, can be condensed, the engine itself will perform additional work, roughly in proportion to the completeness of the condensation that takes place. As mentioned in the former article, by far the larger portion of the heat that is required to produce steam is "latent" in the steam; and if the steam can be converted into water it gives up that latent heat. It is for this reason that steam is such a convenient agent for delivering heat to different parts of a building.

### Oil Separators.

Hard water is often encountered in hospital work. Small quantities of the oil, necessary for lubricating the steam-engines, may be carried into the condenser, and sometimes back into the boiler. The oil is eliminated by apparatus called "Oil Separators," in which the difference in the weight of the oil and the steam, or the water, is made to cause the oil to separate itself out; it is then conducted away to a receptacle provided for it, and used over again after filtration.

[The first article appeared on May 30, page 244.]

